# Tracing human influence on rising surface air temperature in Venezuela

**DOI:** 10.1038/s41598-024-79671-x

**Published:** 2024-11-14

**Authors:** Basudev Swain, Marco Vountas, Aishwarya Singh, Sachin S. Gunthe

**Affiliations:** 1 Institute of Environmental Physics, University of Bremen, Bremen, Germany; 2 Department of Civil Engineering, Indian Institute of Technology Madras, Chennai, India; 3 Centre for Atmospheric and Climate Sciences, Indian Institute of Technology Madras, Chennai, India

**Keywords:** Climate sciences, Climate change, Climate-change impacts

## Abstract

The rise in surface air temperature (SAT) in Venezuela, leading to the loss of all its glaciers, underscores the urgency of understanding human contributions to this phenomenon. This study investigates the impact of anthropogenic climate forcings on SAT across Venezuela, employing observational data, multi-model simulations, and optimal fingerprinting method. Anthropogenic forcings have driven a 0.40–0.85 $^{\circ }$C SAT rise during the industrial era, with land use (LU) emerging as a significant driver (0.36–0.68 $^{\circ }$C), surpassing greenhouse gases (GHGs) (0.10–0.62 $^{\circ }$C). Conversely, anthropogenic aerosols (Aaer) exhibit a cooling effect (− 0.93 to − 0.25 $^{\circ }$C) on SAT. Projections under Representative Concentration Pathways 4.5 indicate substantial SAT increases by the 21st century’s end, underscoring human-induced SAT rise. Effective management of regional Aaer and LU changes in Venezuela holds the potential for mitigating current and future warming and its subsequent impacts on the fragile ecosystem of this region.

## Introduction

The impact of climate change induced by the rise in surface air temperature (SAT) is a profound and pressing global concern^1,2^. Among the most visible and alarming manifestations of this phenomenon is the rapid retreat and eventual disappearance of glaciers^3–5^. Venezuela has become a striking example of this environmental crisis, between 1952 and 2019 alone, Venezuela’s glacier surface went from 2.317 square kilometers to just 0.046 square kilometers^6,7^. Venezuela has experienced the loss of its glaciers as an extreme consequence of climate change, making it the first country in the 21st century to lose its glaciers completely^6^. This event underscores the far-reaching consequences of rising temperatures and highlights every increase in temperature matters. This emphasizes the urgent need for comprehensive climate action to mitigate further losses and protect the local fragile ecosystems.

SAT influenced by various forcings, is instrumental in the glacier melting amid unprecedented global and regional warming^3,8^. Furthermore, the recent few decades have experienced significantly higher warming compared to the pre-industrial period^9^. Global SAT was 1.09 $^{\circ }$C higher in the recent decade compared to the baseline of 1850–1900^9^, with more substantial increases observed over land (1.59 $^{\circ }$C) than over the ocean (0.88 $^{\circ }$C)^10^. Whereas, each region exhibits distinct temperature variations from the global average^8^ and regional warming is a consequential effect of various regional modifications due to human activities^9^. Consequently, understanding the underlying attribution of human influence on the rise in SAT over the broader region of Venezuela, becomes increasingly crucial. Thus, this article primarily focuses on assessing human influence on the rapid increase in SAT in Venezuela.

The impact of human influence on rising SAT during the industrial period, relative to the pre-industrial period, can be evaluated using simulations from the Coupled Model Intercomparison Project Phase 5 (CMIP5)^11^. CMIP5 is supported by the World Climate Research Program (WCRP) and presents simulations with different forcings by using different climate models of various spatio-temporal scales. These different simulations are considered in various reports on climate change such as the Intergovernmental Panel on Climate Change (IPCC) Assessment Reports AR4 and AR5^10^, cover historical periods (19th, and 20th century) and projections for the future.

The future projections (21st century) under different Representative Concentration Pathways (RCPs)^12^. RCPs show various future scenarios based on estimated future emissions of greenhouse gases (GHG), aerosols, ozone, and land-use changes. CMIP5 experiments simulate historical temperature variations driven by factors such as anthropogenic aerosols (Aaer), greenhouse gases (GHGs), land use change (LU), and natural forces (NAT) (solar irradiance + volcanic activity)^12^. By comparing these forcings, we can better understand the factors contributing to climate change in specific regions or locations^9^.

To isolate the impact of human influence on the increase in SAT, optimal detection and attribution technique^13^ have been extensively used^9,14^. This study uniquely focuses on evaluating the human impact on SAT in the broader area encompassing Venezuela, along with small portions of adjacent regions in Colombia and Brazil (henceforth called *study region*). In order to discern human influence, we estimate the warming relative to pre-industrial levels of SAT. Despite various Holocene epochs that may be used as pre-industrial reference periods, as highlighted by^15^, we follow the guidelines set by the IPCC Special Report on Global Warming of 1.5 $^{\circ }$C^16^, using the chosen base period (pre-industrial) of 1850–1900. This provides a more pertinent approach to monitoring human influence on SAT in Venezuela during the recent (1955–2020) and future industrial period (2020-2100)^16^.

## Results

### Temporal evolution of human influence on rise in SAT

Due to the good agreement between the observational datasets, such as HadCRUT5^17^, ERA5^18^ and various CMIP5 historical model simulations across the study region over the last 50 years (see “Methods” section, Fig. S1), we further analysed the contributions of individual forcings to the SAT over the study region.

**Figure 1:**
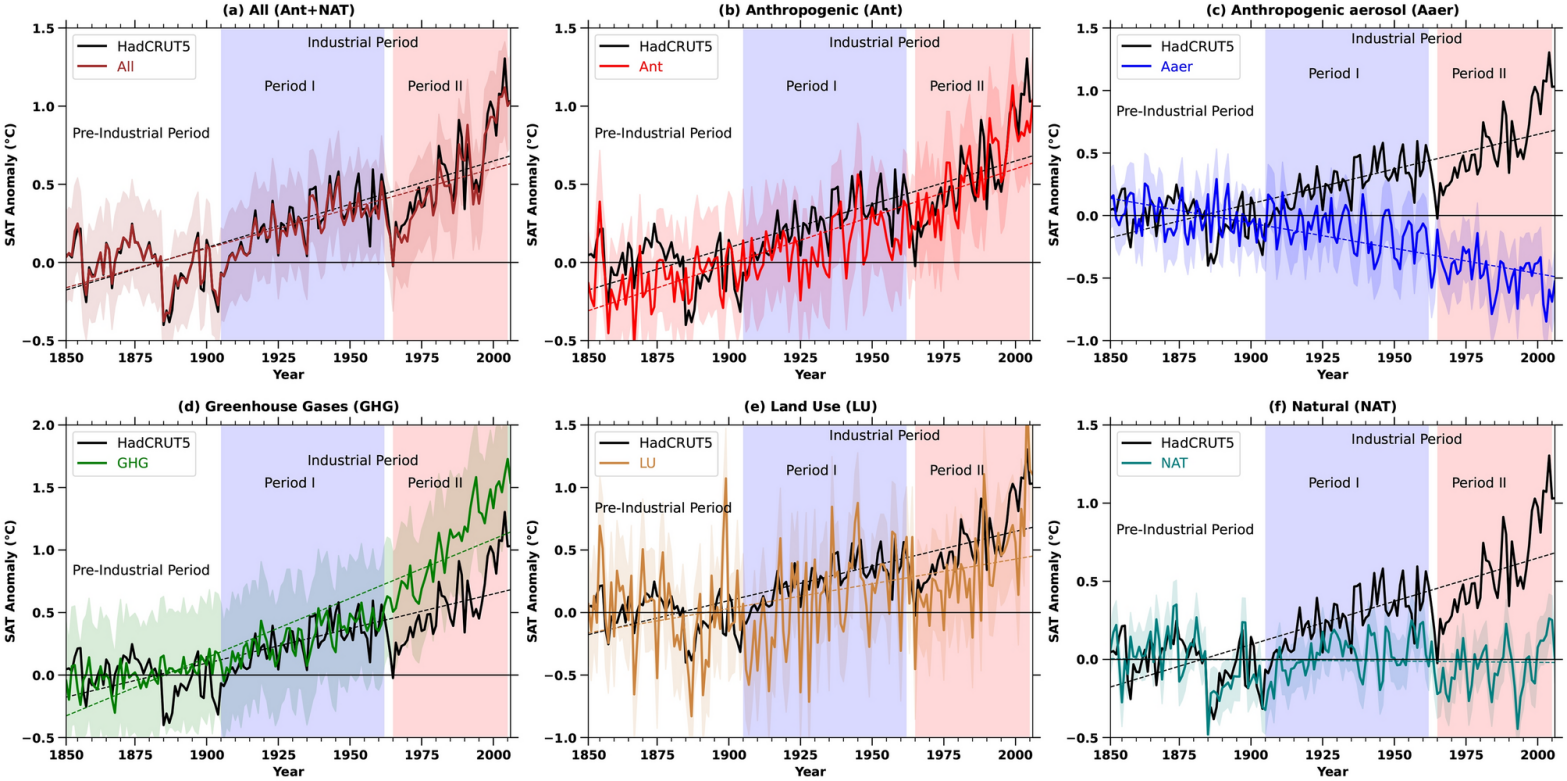
Temporal evolutions of each forcing and attribution of human influence over Venezuela. The graph illustrates the temporal variation of annual surface air temperature anomalies from 1850 to 2005. The category “All” encompasses historical forcings, representing all types of influences. Specific forcings such as anthropogenic (Ant = All-NAT), Aaer, GHG, LU, Natural forcings (NAT) (this includes solar radiation and volcanic eruptions), and Anthropogenic forcings (Ant) are individually depicted. Additionally, observed HadCRUT5 temperature data for the period 1850–2005 is included for each forcing. Robust regression analysis was conducted at a 95% confidence level to analyze trends, and the results are superimposed in each panel. Only the regression lines that have a 95% confidence level are presented.

We presented the ensemble averages of various historical forcings, such as anthropogenic (Ant), greenhouse gases (GHG), anthropogenic aerosols (Aaer), land use (LU), and natural (NAT) for the 19th and 20th centuries, as detailed in Table S1 and Fig. 1a–f. These temporal changes are computed as the annual mean for the study region (from January 1850 to December 2005) minus the annual mean SAT values of each simulation for the pre-industrial period (1850–1900). Each panel includes shaded regions indicating the ensemble bounds (maximum and minimum values) along with regression fits.

**Table 1:** Optimal fingerprinting estimations of each forcing to the change in surface air temperature.

	NAT	Ant	Aaer	GHG	LU
(a) Industrial Period (1955–2005) ($^{\circ }$C)					
HadCRUT5	0.02 (− 0.04 to 0.08)	0.62 (0.40 to 0.85)	− 0.59 (− 0.93 to − 0.25)	0.36 (0.10–0.62)	0.52 (0.36–0.68)
(b) Entire Future Period (2006–2100) ($^{\circ }$C)					
		0.76 (0.09 to 0.43)	0.53 (− 0.46 to 1.53)	0.62 (0.20 to 1.04)	
(c) Near Future (2010–2040) ($^{\circ }$C)					
		0.04 (− 0.37 to 0.46)	0.11 (− 0.28 to 0.52)	0.53 (0.23 to 0.84)	
(d) Mid Future (2040–2070) ($^{\circ }$C)					
		0.13 (− 0.10 to 0.38)	− 0.01 (− 0.28 to 0.25)	0.12 (− 0.11 to 0.36)	
(e) Far Future (2070–2100) ($^{\circ }$C)					
		0.02 (− 0.04 to 0.10)	0.03 (− 0.03 to 0.10)	0.05 (− 0.01 to 0.11)	

The table presents multimodel estimates of attributable temperature change in degrees Celsius between the periods 1955–2005, and 2006–2100, with respect to 1850–1900. It includes $\beta$ as the vector of coefficients, and 5–95% confidence intervals in brackets for attributable warming. (a) The attributable warming is derived by using HadCRUT5 observational data and various forcings, such as Natural Forces (NAT), Anthropogenic Aerosols (Ant), Aaer, Greenhouse Gases (GHG), and Land Use (LU) signals. (b) The attributable warming is derived from the RCP4.5 analysis for various forcings, such as Anthropogenic Aerosols (Aaer), and Greenhouse Gases (GHG) signals for entire future period. (c, d, and e) The attributable warming is derived from the RCP4.5 analysis for various forcings for near, mid, and far future respectively.

Figure 1a clearly illustrates the transition of anomalies, which fluctuate between negative and positive values from 1850 to 1900, followed by predominantly positive values thereafter. The year 1900 signifies a shift from negative to positive temperature anomalies in the annual SAT variation, which closely corresponds with the historical ensemble mean of the model simulations and the HadCRUT5 observations. All other forcings (Ant, GHG, LU) show a remarkable increase in SAT after 1900 (Fig. 1b,d,e), except for Aaer and NAT (Fig. 1c,f). GHG forcing leads to even higher forcing than the cumulative impact of all forcings (Fig. 1d). This suggests that considering GHG and LU alone leads to a high estimation of increase in SAT over the study region during the industrial period (Fig. 1d,e).

During the beginning of the industrial period (Period I, from 1900 to 1955), the warming due to LU shows an increase after 1900 over the study region then it shortly declined and rapidly increased from 1960 (Period II, 1960 to 2005) (Fig. 1e), which is consistent with the observation and historical simulations (Fig. 1a). The effect of LU can also be seen in the anthropogenic SAT anomaly after 1960, during the second half of the industrial period (Period II) (Fig. 1b). Further, Fig. 1b indicates that the CMIP5 historical simulations exhibit minimal net anthropogenic warming prior to 1925.

The noisiness in SAT responses due to land use (LU) forcing compared to other forcings likely stems from the localized and heterogeneous nature of land use changes. Unlike global-scale forcings such as greenhouse gases or aerosols, which tend to have more uniform effects on temperature, LU forcings are spatially variable, affecting different regions in diverse ways based on changes in vegetation, albedo, soil properties, and evapotranspiration. This regional variability can lead to localized heating or cooling, which, when aggregated globally, produces a noisy or less smooth SAT response.

Moreover, land use changes can amplify or dampen temperature variability due to interactions with local climate processes, such as soil moisture feedbacks and shifts in atmospheric circulation patterns, which introduce additional variability into the SAT response to LU forcing. This stands in contrast to more uniform, globally distributed forcings, which tend to produce smoother SAT changes over time^19^.

**Figure 2:**
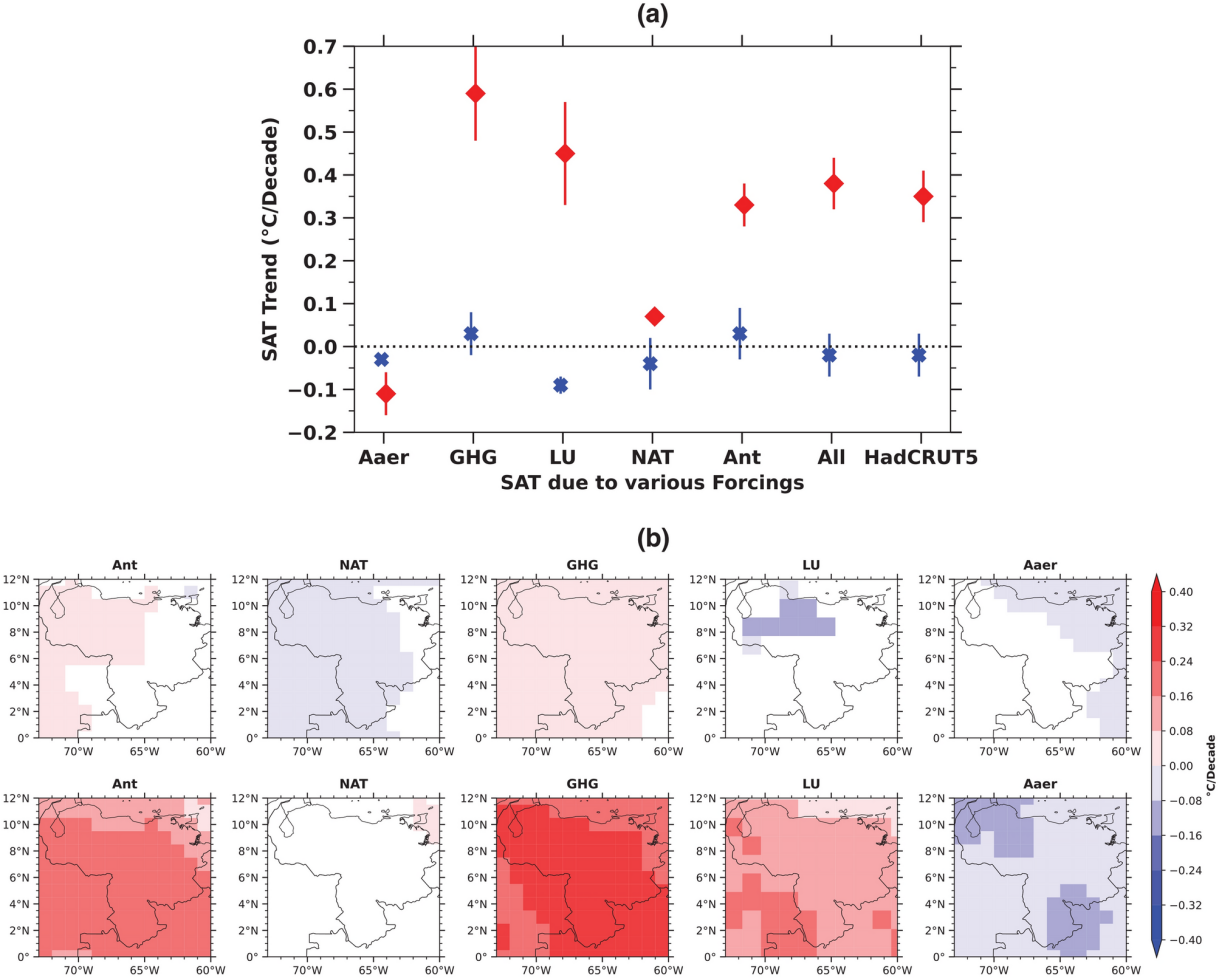
Pre-industrial (from 1850 to 1900) and Industrial period (from 1955 to 2005) SAT trends over Venezuela. (**a**) The average trends of various forcings are presented as the pre-industrial period, and industrial period as blue and red respectively. (**b**) The SAT trends presented spatially for various forcings over Venezuela is presented as, (top row) for the pre-industrial period, (bottom row) for the industrial period. Insignificant trends are masked out.

This suggests that there was minimal net anthropogenic impact on SAT during the early twentieth century, with most anthropogenic warming occurring post-1960. Land use (LU) changes and greenhouse gases (GHG) have had a significant influence (Fig. 1b,d,e). The interplay of direct and indirect anthropogenic aerosol (Aaer) forcings shows a marked decline in SAT starting from the 1960s (Period II) (Fig. 1c).

Thus the forcing due to Aaer played a counterbalancing role to the increase in Land use and land cover change during the industrial period. When looking at the temporal development, the impact of natural forcings on SAT is comparatively smaller than those from GHG and LU (Fig. 1f). In summary, the findings indicate that Aaer exerted a notable cooling influence, thereby decelerating the rate of warming (Fig. 1c). Conversely, other forcings such as greenhouse gases (GHG) and land use (LU) changes substantially contributed to the observed rise in SAT (Fig. 1d,e).

We further examined the spatial attribution of the individual forcings relevant in the study region. We segmented the forcings into individual components as SAT trends (Fig. 2a) and spatial distribution (Fig. 2b) for both the pre-industrial and industrial periods. Observational SAT data (HadCRUT5, and ERA5) and multi-model mean indicate that a substantial portion of the north-western area of Venezuela, where most of the glaciers were present, experienced accelerated warming due to LU (0.21 $^{\circ }$C/decade) and GHG forcings (0.26 $^{\circ }$C/decade) during the industrial era (Fig. 2b, second row) compared to the pre-industrial period (Fig. 2b, first row). Similar spatial patterns of temperature trends are observed across different observational datasets such as HadCRUT5, ERA5, and the multi-model mean (Fig. S2).

Over the study region, SAT trends have increased during the industrial period due to Ant, NAT, Aaer, GHG, and LU forcings, ranging from 0.03 to 0.33, − 0.04 to − 0.07, − 0.03 to − 0.11, 0.03 to 0.59, and − 0.09 to 0.45 $^{\circ }$C/decade, respectively (Fig. 2a). This underscores the higher impact of human activities to the rise in SAT, particularly in terms of GHG, LU forcings, and Aaer forcing induced a cooling effect during the industrial period. Additionally, NAT forcing had a negligible impact (Fig. 2b). The SAT increase due to LU forcings over the study regions during the industrial period can therefore be attributed to the impact of human activities (Fig. 2b), consistent with the observations (Fig. S2).

### Optimal fingerprinting for tracing human influence

Using a multimodel average of all 22 models (Table S1), as well as observational data from HadCRUT5, alongside the optimal fingerprinting method^13^, we determined the contributions of various forcings to SAT changes in this region (Table 1a). Our analysis reveals that 0.40–0.85 $^{\circ }$C (5–95% range) of increase in SAT over the study region during the industrial period relative to the pre-industrial period can be attributed to anthropogenic forcings. Among these forcings, Aaer, GHG, and LU contribute changes of − 0.93 to − 0.25 $^{\circ }$C, 0.10–0.62 $^{\circ }$C, and 0.36–0.68 $^{\circ }$C, respectively, with LU forcings showing the highest contribution to the overall SAT increase over study region (Table 1a), followed by GHG, while NAT forcings have a negligible impact of − 0.04 to 0.08 $^{\circ }$C. Notably, SAT over the study region is primarily driven by ANT forcing, with the highest contribution from LU forcings followed by GHG in the industrial period.

**Figure 3:**
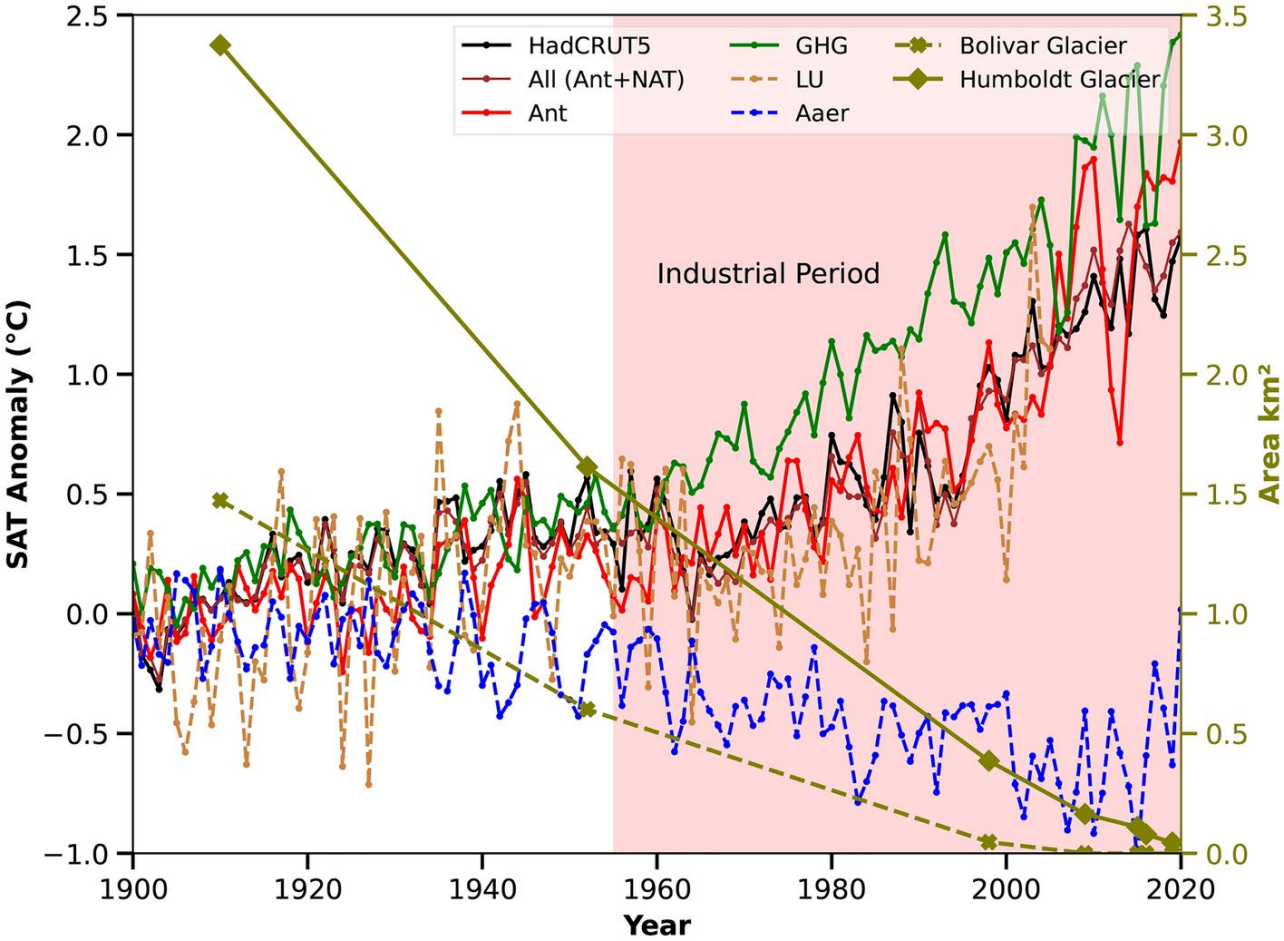
Rise in SAT due to various anthropogenic and natural forcings and the loss of glaciers during the industrial period over Venezuela. The anomaly of total SAT from the model and HadCRT5 as well as SAT anomaly due to various anthropogenic and natural forcings are presented The trend of various forcings, together with the Bolivar and Humboldt glacier area in km$^2$ are shown for the industrial period are presented. The Bolivar Glacier completely melted in 2011, whereas the Humboldt Glacier melted in 2019. The CMIP5 data is available from 1850 to 2005, so for the time period of 2006 to 2020, the total SAT, as well as the various forcings (GHG, Aaer, NAT), are taken from CMIP6 scenario SSP245, whereas the LU forcing is available from 1850 to 2005 form CMIP5 simulations.

To assess the impact of human-driven SAT rise detected by the optimal fingerprinting method on Venezuela’s status as the first glacier-free nation in the 21st century, we analyzed glacier area changes in the Bolivar and Humboldt Glaciers. Historically, Venezuela had six glaciers in the Sierra Nevada de Mérida range at around 5,000 meters altitude above sea level^6,7^. By 2011, only Humboldt Glacier remained^6,7^. Over the industrial period, glacier loss accelerated by 98% (Fig. 3), with regional temperature trends rising from − 0.02 $^{\circ }$C to 0.35 $^{\circ }$C per decade. Human activities, particularly land use changes (0.36–0.68 $^{\circ }$C) and greenhouse gases (0.10–0.62 $^{\circ }$C), were the primary drivers of SAT rise and eventual loss of all of its glaciers (Fig. 3).

### Future projections

**Figure 4:**
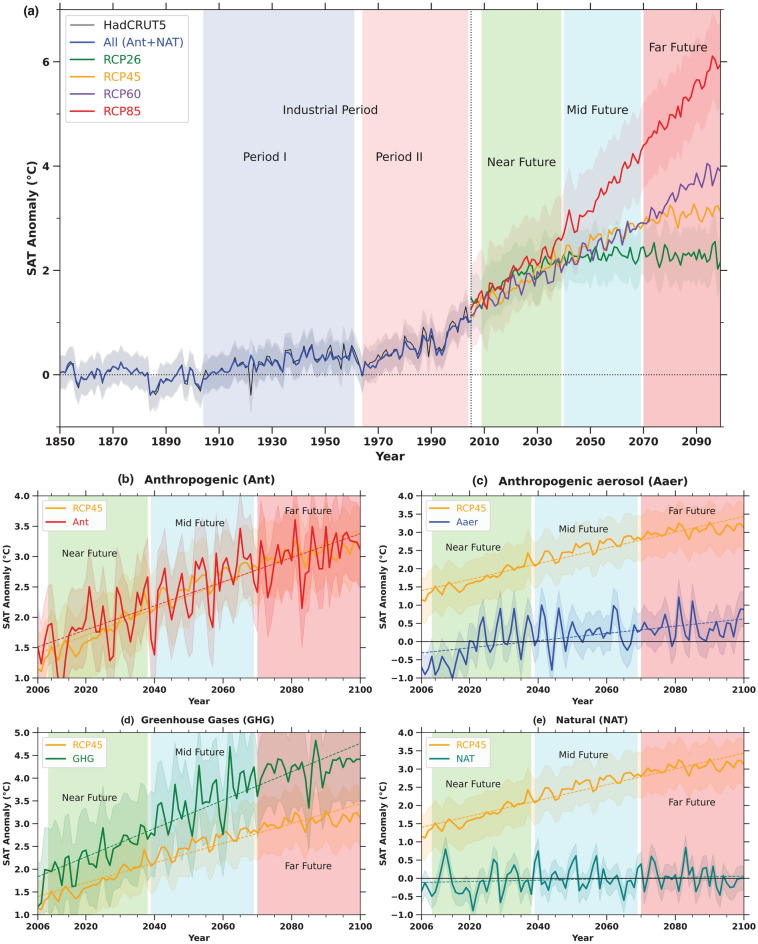
The past, present, and projected future trends in surface air temperature over Venezuela are depicted in the graph. It shows the annual mean surface air temperature is calculated by averaging data from different climate models simulations from 1850 to 2100, relative to the pre-Industrial period of 1850–1900. (**a**) Future projection scenarios from various Representative Concentration Pathways (RCPs) are represented for the period 2006–2100. The shaded region indicates the standard deviation of the ensemble mean of CMIP5 models. Additionally, observed data sets from HadCRUT5 (1850–2005) are plotted in black and red, respectively. (**b**–**e**) The future projections of Anthropogenic, Anthropogenic Aerosols, Greenhouse gases, and Natural forcings respectively under SSP245 of CMIP6, which is similar to RCP4.5 of CMIP5.

An increase in SAT over the study region from the combination of all forcings (historical) closely follows the observed HadCRUT5 historical temperatures throughout the pre-industrial and industrial periods (Fig. 4a). Given the agreement observed between the observational data and the multi-model mean, we have gained confidence in projecting the SAT increase across the region through the 21st century. Examining future projections is crucial despite the irreversible loss of glaciers in the region, but limiting anthropogenic activities offers a potential solution to mitigate the further rise in SAT and their subsequent effects on the delicate ecosystems of the study area.

Our projections encompassed both low and high Representative Concentration Pathways (RCPs) emission scenarios, ranging from RCP2.6 to RCP8.5. By the century’s end, RCP2.6 and RCP8.5 scenarios exhibit temperature anomalies of approximately 2.1 $^{\circ }$C and 6 $^{\circ }$C, respectively, over the study area (Fig. 4a). Trends in SAT projections indicate a consistent rise under RCP8.5, whereas mean temperatures from RCP2.6 exhibit an initial rise until 2050, followed by a slight decline, aligning with the radiative forcing trends associated with RCP2.6 (Fig. 4a).

According to studies^20,21^, we are currently on track with the RCP4.5 pathway, which represents a middle-ground scenario between the lowest (RCP2.6) and highest (RCP8.5) projections. Under RCP4.5, SAT is expected to rise by as much as 3.2 $^{\circ }$C by the year 2100 (Fig. 4a). To better understand the future temperature increase, we examined the impact of different human-driven factors under the Shared Socioeconomic Pathway (SSP245)^22^ scenario, based on CMIP6 models^11^. This is because CMIP5 simulations only provide projections for overall SAT rise and lack detailed information on specific forcings for future^11,23^. The SSP245 scenario aligns with RCP4.5 from CMIP5^11,23^, but CMIP6 data does not yet offer projections on temperature changes due to individual land use (LU) factors in future scenarios^11^.

To accurately determine the role of human activities in future SAT increases using the optimal fingerprinting method under the RCP4.5 scenario, we divided the projections into distinct time frames: the near future (2010–2040), mid-century (2040–2070), and far-future (2070–2100). Additionally, we analyzed the full timeline of model simulations from 2010 to 2100 for a comprehensive assessment (Fig. 4b–e), and (Table 1b–e).

Our analysis shows that a 0.09 to 0.43 $^{\circ }$C rise in SAT across the study area from 2010 to 2100, compared to the pre-industrial period, is due to human-induced factors (Table 1b). Anthropogenic aerosols (Aaer) and greenhouse gases (GHG) contribute to temperature changes ranging from − 0.46 to 1.53 $^{\circ }$C and 0.20 to 1.04 $^{\circ }$C, respectively. Interestingly, the warming effect from aerosols is projected to be on par with GHG impacts over this period (Table 1b). While aerosols had a cooling effect during the industrial period (− 0.93 to − 0.25 $^{\circ }$C) (Table 1a), they are expected to cause warming in the future, ranging from 0.46 to 1.53 $^{\circ }$C (Table 1b). This shift is likely due to reduced aerosol emissions as clean air policies lower the scattering of solar radiation^24,25^. The most significant warming from both anthropogenic aerosols and GHG is projected to occur in the near future (Table 1c) and (Fig. 4c,d), with values between − 0.28 to 0.52 $^{\circ }$C for aerosols and 0.20 to 1.04 $^{\circ }$C for GHG, before stabilizing in the mid and late future. This pattern aligns with the RCP4.5 scenario, which forecasts a sharp temperature increase in the near term followed by relative stabilization in later decades, as depicted in Fig. 4a–e.

## Discussion

Detection and attribution analyses offer insights into how various factors have contributed to the observed increase in surface air temperature (SAT) across Venezuela, and nearby border regions, such as parts of Colombia and Brazil (the study region). It further sheds light on their impact on SAT changes. This information is crucial for understanding the influence of human activities amid increasing industrialization and evolving land use and land cover dynamics on SAT to mitigate the climate change impacts. While future climate change is anticipated to be predominantly driven by Greenhouse Gas (GHG) changes, an often overlooked aspect is land use (LU) forcing.

Our findings reveal notable contributions from LU forcings, ranging from 0.36 to 0.68 $^{\circ }$C over the study region followed by GHG forcings (0.10–0.62 $^{\circ }$C) (Table 1) to the overall increase in SAT. Our results underscore the substantial influence of human-induced changes in LU, anthropogenic aerosols, and GHG during the industrial period has made Venezuela the first country in the 21st century to lose all of its glaciers (Fig. 3). Venezuela’s loss of its last glacier (the Humboldt glacier) marks a sad milestone, but it also offers a critical chance to highlight the urgent impacts of regional climate change. It presents an opportunity to develop strategies to protect high-altitude ecosystems from further damage in South American Andean countries. In an attempt to slow the glacier melting, this study emphasizes that the pressing need now is to limit the effects of regional human activities on rising temperatures (Fig. 3). Gaining a clear understanding of this connection is essential for devising measures to curb regional climate change. Without immediate action, Venezuela’s glacier loss could signal a broader regional trend in the South American Andean countries, putting vital water sources and ecosystems at risk. This event highlights the necessity for swift intervention to reduce human-induced regional factors, such as land use changes, greenhouse gas emissions, and anthropogenic aerosols to curb the future increasing SAT and its consequential effects on Venezuela’s delicate ecosystems amid the increasing human activities during the ongoing industrial era.

In this study, we utilized a single ensemble (r1i1p1) because the SAT data for different forcings are only available for this specific ensemble^23^. To ensure consistency, we used the same ensemble for both the total simulated SAT and the SAT associated with various forcings for historical and future projections. However, as highlighted by^26^, while single-model ensembles have significant value, using a multi-model collection of large ensembles offers a more robust approach. This allows for a better comparison of both forced responses on regional or decadal scales and internal variability across models. It also enhances model evaluation by providing comprehensive insights into biases, distinguishing those caused by internal variability from those related to forced responses. Unlike CMIP^11,23^, a multi-model data storage of large ensembles enables direct separation of projection uncertainty into components because of the variability internally. Although this approach has clear benefits, most studies^9,27–30^ have relied on only one ensemble due to the complexity of accessing vast amounts of data from various sources.

## Method

In this study, an analysis was conducted on 158 simulations derived from 22 CMIP5^11^ models, encompassing various emission scenarios and historical simulations, as detailed in Table S1. Notably, not all CMIP5 models incorporate all forcings. The simulations encompass 22 CMIP5 models across diverse emission scenarios^12^, including RCP 2.6, 4.5, 6.5, and 8.5, along with NAT forcings from 17 simulations, GHGs forcings from 18 simulations, Aaer forcings from 10 simulations, and LU forcings from 5 simulations. The historical period from 1850 to 2005, designated for coordinated climate model experiments under CMIP5, was utilized for simulation. These experiments, part of the CMIP5 coordinated experiments utilized in the IPCC’s Fifth Assessment Report^31^, involve reproducing historical climate by varying atmospheric composition resulting from solar forcing, anthropogenic and volcanic emissions, and natural and anthropogenic aerosols. Multiple ensembles from 22 CMIP5 models were employed, offering monthly mean SAT outputs for all four scenarios (RCP2.6, RCP4.5, RCP6.0, and RCP8.5), as well as historical simulations with different forcings.

Further, to better understand the future temperature increase, we examined the impact of different human-driven factors under the Shared Socioeconomic Pathway (SSP245) scenario, based on CMIP6 models from 2006 to 2100. This is because CMIP5 simulations only provide projections for overall surface temperature rise and lack detailed information on specific forcings for the future. The SSP245 scenario of CMIP6 aligns with RCP4.5 from CMIP5, but CMIP6 data does not yet offer projections on temperature changes due to individual land use (LU) factors in future scenarios. The same models are selected from CMIP6 as well as CMIP5, which is presented in Table S1.

In this study, we utilized a single ensemble (r1i1p1) from CMIP5 and CMIP6, because the SAT data for different forcings are only available for this specific ensemble in CMIP5. To ensure consistency, we used the same ensemble for both the total simulated SAT and the SAT associated with various forcings for historical and future projections from CMIP5 and CMIP6.

To further refine the analysis, Taylor diagrams were employed (Fig. S1a,b), showcasing simulated mean SATs relative to observational data from HadCRUT5^17^ and ERA5^18^ for the period 1955–2005 over Venezuela. This industrial period timeframe is chosen for model evaluation due to more availability of ground-based and space-borne datasets. Models demonstrating correlations below 0.5 with HadCRUT5 datasets were excluded, leaving fifteen models with high correlations for subsequent analysis. The Taylor diagram illustrates the spread of models concerning correlation, with consistent correlations observed between models and the observational datasets.

Observed SAT was derived from HadCRUT5 monthly anomalies by area weighting and subtracting the long-term mean (1850–1900) to yield anomalies. The median dataset was utilized for the primary analysis results, with each ensemble dataset member treated similarly to derive uncertainties in the multimodel attributable-warming estimates.

SAT data from different forcings and CMIP5 model datasets under various RCPs were standardized to a common grid of $1^{\circ } \times 1^{\circ }$ longitude and latitude using the bilinear interpolation provided by Climate Data Operators (CDO)^32^. Robust regression, based on Iteratively Reweighted Least Squares Regression^33^, was employed for trend estimation to accommodate outliers, with the t-test analysis used to determine the statistical significance of temperature trends. Spectral peaks with confidence levels exceeding 95% were considered.

### Optimal fingerprinting method

Furthermore, an optimal detection analysis was conducted utilizing the regularized optimal fingerprinting algorithm^13^ implemented in Python^34^. This method assesses the impact of each forcing on observed changes in climate variables, such as temperature^9,13,35–37^.

The mathematical steps involved in the Optimal Fingerprinting method are as follows;

**Linear Regression Model:** Assume that the total SAT changes ($y$) can be expressed as a linear combination of different external forcings ($x_1, x_2, \ldots , x_n$) plus a residual term ($\varepsilon$):

$$\begin{aligned} y = \beta _0 + \beta _1 x_1 + \beta _2 x_2 + \ldots + \beta _n x_n + \varepsilon \end{aligned}$$

**Matrix Formulation:** Organize the data into matrices. Let $X$ be the matrix of SAT due to various forcings, such as Ant, GHG, LU, Aaer, NAT (including a column of ones for the intercept term), $Y$ be the vector of total SAT from HadCRUT5 observation, and $\beta$ be the vector of coefficients to be estimated:

$$\begin{aligned} X= & \begin{bmatrix} 1 & x_{1,1} & x_{2,1} & \ldots & x_{n,1} \\ 1 & x_{1,2} & x_{2,2} & \ldots & x_{n,2} \\ \vdots & \vdots & \vdots & \ddots & \vdots \\ 1 & x_{1,m} & x_{2,m} & \ldots & x_{n,m} \end{bmatrix}\\ Y= & \begin{bmatrix} y_1 \\ y_2 \\ \vdots \\ y_m \end{bmatrix}\\ \beta= & \begin{bmatrix} \beta _0 \\ \beta _1 \\ \vdots \\ \beta _n \end{bmatrix} \end{aligned}$$

The linear regression equation in matrix form is $Y = X \beta + \varepsilon$.

**Least Squares Estimation:** Estimate the coefficients ($\beta$) using the least squares method, which minimizes the sum of squared differences between the observed and modeled temperatures: $[ \beta = (X^T X)^{-1} X^T Y ]$

**Uncertainty Estimation:** Calculate the residuals ($\varepsilon$), representing the differences between the observed and modeled temperatures. The standard deviation of residuals ($std\_residuals$) is a measure of uncertainty in the model.

**Confidence Interval Calculation:** Compute 95% confidence intervals for each coefficient ($\beta _i$):

$$\begin{aligned} \text {Confidence Interval} = [\beta _i - 1.96 \cdot std\_residuals, \beta _i + 1.96 \cdot std\_residuals] \end{aligned}$$

**Assessment of Significance:** Evaluate the statistical significance of each forcings contribution by examining whether the confidence interval for its coefficient includes zero. If the interval does not include zero, the forcing is considered statistically significant in contributing to the observed temperature changes.

## Supplementary Information

Supplementary Information.

## Data Availability

All the CMIP5 model datasets are available at https://esgf-data.dkrz.de/search/cmip5-dkrz/. Observational data from HadCRUT5 is available at https://crudata.uea.ac.uk/cru/data/temperature/. ERA5 data is available at https://cds.climate.copernicus.eu/cdsapp#!/dataset/reanalysis-era5-single-levels?tab=overview. The galcier data over Venezuela is available at https://www.tandfonline.com/doi/full/10.1080/15230430.2020.1822728#d1e385.

## Code availability

The optimal detection analysis was conducted utilizing the regularized optimal fingerprinting algorithm^13^ is available at http://www.umr-cnrm.fr/spip.php?article23&lang=en.
