# Supplementary material for: Tracing human influence on rising surface air temperature in Venezuela
Source: Sci Rep. 2024 Nov 14;14:28005. doi: 10.1038/s41598-024-79671-x (PMC11564689; doi:10.1038/s41598-024-79671-x)
Supplement: Supplementary file 1 — Supplementary Information. [file 41598_2024_79671_MOESM1_ESM.pdf]

Tracing human influence on rising surface air temperature in Venezuela

Basudev Swain[1,*], Marco Vountas[1], Aishwarya Singh[2,3], and Sachin S. Gunthe[2,3]

[1]Department of Physics, Institute of Environmental Physics, University of Bremen, Germany
[2]Department of Civil Engineering, Indian Institute of Technology Madras, India
[3]Centre for Atmospheric and Climate Sciences, Indian Institute of Technology Madras, India
[*]Correspondence: basudev@iup.physik.uni-bremen.de

ABSTRACT

The rise in surface air temperature (SAT) in Venezuela, leading to the loss of all its glaciers, underscores the urgency of understanding human contributions to this phenomenon. This study investigates the impact of anthropogenic climate forcings on SAT across Venezuela, employing observational data, multi-model simulations, and optimal fingerprinting method. Anthropogenic forcings have driven a 0.44 to 0.90℃ SAT rise during the industrial era, with land use (LU) emerging as a significant driver (0.40 to 0.71℃), surpassing greenhouse gases (GHGs) (0.09 to 0.65℃). Conversely, anthropogenic aerosols (Aaer) exhibit a cooling effect (-0.96 to -0.24℃) on SAT. Projections under Representative Concentration Pathways 4.5 indicate substantial SAT increases by the 21st century's end, underscoring human-induced SAT rise. Effective management of regional Aaer and LU changes in Venezuela holds the potential for mitigating current and future warming and its subsequent impacts on the fragile ecosystem of this region.

Sl.No.	Model Name	Historical (1850-2005)					Future Projection (2006-2100)			
		Historical	AA	GHG	LU	NAT	RCP2.6	RCP4.5	RCP6.0	RCP8.5
1	bcc-csm1-1	Y	N	Y	N	Y	Y	Y	Y	Y
2	BNU-ESM	Y	N	Y	N	Y	Y	Y	N	Y
3	CanESM2	Y	Y	Y	Y	Y	Y	Y	Y	Y
4	CCSM4	Y	Y	Y	Y	Y	Y	Y	Y	Y
5	CESM1-CAM5	Y	N	Y	N	N	Y	Y	Y	Y
6	CNRM-CM5	Y	N	Y	N	Y	Y	Y	Y	Y
7	CSIRO-MK3	Y	Y	Y	N	Y	Y	Y	Y	Y
8	FGOALS_g2	Y	Y	Y	N	Y	Y	Y	N	Y
9	GFDL-CM3	Y	Y	Y	N	Y	Y	Y	Y	Y
10	GFDL-ESM2M	Y	Y	Y	Y	Y	Y	Y	Y	Y
11	GISS-E2-H	Y	Y	Y	Y	Y	Y	Y	Y	Y
12	GISS-E2-R	Y	Y	Y	Y	Y	Y	Y	Y	Y
13	HadGEM2-CC	Y	N	N	N	N	Y	Y	Y	Y
14	HadGEM2-ES	Y	N	Y	N	Y	Y	Y	Y	Y
15	inmcm4	Y	N	N	N	N	Y	Y	Y	Y
16	IPSL-CM5A-LR	Y	Y	Y	N	Y	Y	Y	Y	Y
17	IPSL-CM5A-MR	Y	N	Y	N	Y	Y	Y	Y	Y
18	MIROC-ESM	Y	N	Y	N	Y	Y	Y	Y	Y
19	MIROC5	Y	N	N	N	N	Y	Y	Y	Y
20	MPI-ESM-LR	Y	N	N	N	N	Y	Y	Y	Y
21	MRI-CGCM3	Y	N	Y	N	Y	Y	Y	Y	Y
22	NorESM1-M	Y	Y	Y	N	Y	Y	Y	Y	Y
	Total	22	10	18	5	17	22	22	20	22

Table 1. Table containing information about different models used in this study. Our study utilizes 158 CMIP5 simulations encompassing RCP26, RCP45, RCP60, and RCP85 scenarios available for r1i1p1 simulations. The historical simulations incorporate various forcings, with 'Y' indicating the inclusion of a particular forcing and 'N' denoting its absence. The forcings include anthropogenic aerosols (AA), well-mixed greenhouse gases (GHG), land-use change (LU), and natural forcings (NAT). All forcing information was sourced from the CMIP5 website: http://cmip-pcmdi.llnl.gov/index.html.). We have used the CMIP6 model Shared Socioeconomic Pathway (SSP245) future scenario simulations (from 2006 to 2100) for various forcings (such as GHG, Aaer, NAT, but the LU forcing for the future is not available in CMIP6). The same models are selected for CMIP6 as well.

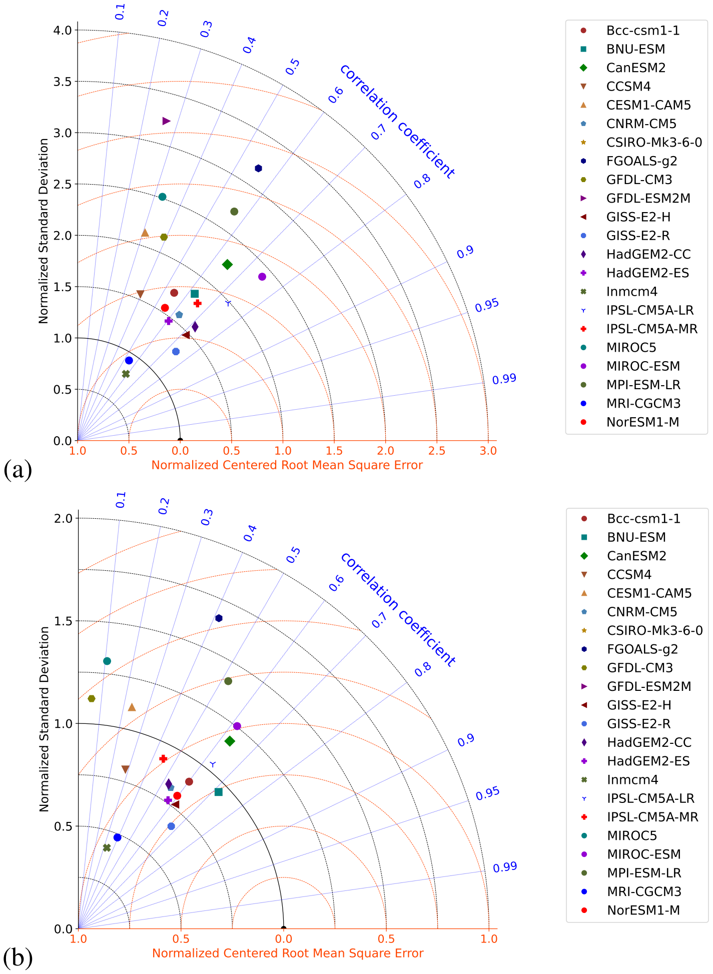

Figure 1. Taylor diagram presenting evaluation of surface air temperature simulated by different models with respect to observations. The Taylor diagram illustrates the mean surface temperature comparison among HadCRUT5, and individual CMIP5 models from 1955 to 2005 (a). Panel (b) shows the comparison between ERA5 and CMIP5 models during the same period. Solid lines represent standard deviations, while dotted lines indicate correlations.

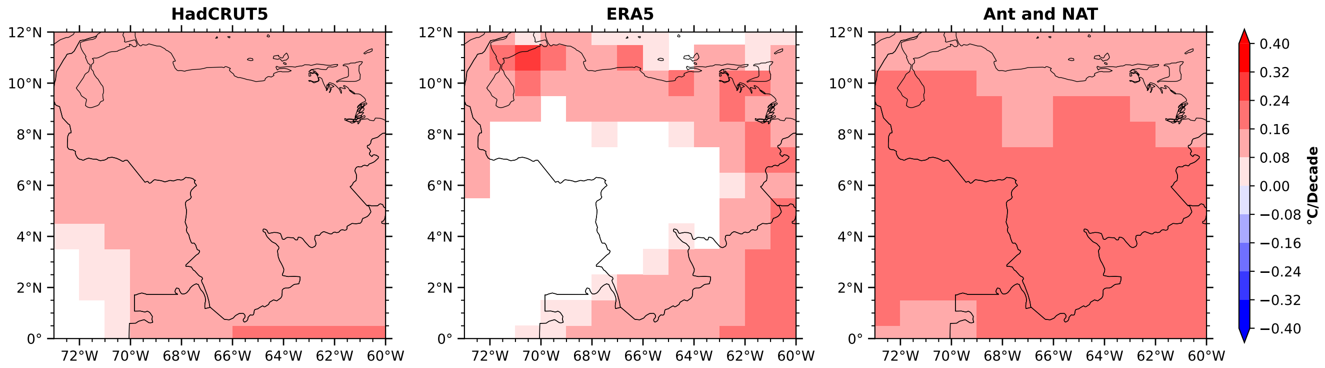

Figure 2. Spatial Surface air temperature trends from HadCRUT5, ERA5, and multi-model mean (Ant+NAT). Temperature trends for the period 1955–2005 are depicted using the average of observational datasets like HadCRUT5 and ERA5. Regions lacking statistically significant changes are masked out.
